# Positive natural selection in primate genes of the type I interferon response

**DOI:** 10.1186/s12862-021-01783-z

**Published:** 2021-04-26

**Authors:** Elena N. Judd, Alison R. Gilchrist, Nicholas R. Meyerson, Sara L. Sawyer

**Affiliations:** Department of Molecular, Cellular and Developmental Biology; BioFrontiers Institute, University of Colorado Boulder, Boulder, USA

**Keywords:** Interferon, Virus, Evolution, Arms race, Positive selection

## Abstract

**Background:**

The Type I interferon response is an important first-line defense against viruses. In turn, viruses antagonize (i.e., degrade, mis-localize, etc.) many proteins in interferon pathways. Thus, hosts and viruses are locked in an evolutionary arms race for dominance of the Type I interferon pathway. As a result, many genes in interferon pathways have experienced positive natural selection in favor of new allelic forms that can better recognize viruses or escape viral antagonists. Here, we performed a holistic analysis of selective pressures acting on genes in the Type I interferon family. We initially hypothesized that the genes responsible for inducing the production of interferon would be antagonized more heavily by viruses than genes that are turned on as a result of interferon. Our logic was that viruses would have greater effect if they worked upstream of the production of interferon molecules because, once interferon is produced, hundreds of interferon-stimulated proteins would activate and the virus would need to counteract them one-by-one.

**Results:**

We curated multiple sequence alignments of primate orthologs for 131 genes active in interferon production and signaling (herein, “induction” genes), 100 interferon-stimulated genes, and 100 randomly chosen genes. We analyzed each multiple sequence alignment for the signatures of recurrent positive selection. Counter to our hypothesis, we found the interferon-stimulated genes, and not interferon induction genes, are evolving significantly more rapidly than a random set of genes. Interferon induction genes evolve in a way that is indistinguishable from a matched set of random genes (22% and 18% of genes bear signatures of positive selection, respectively). In contrast, interferon-stimulated genes evolve differently, with 33% of genes evolving under positive selection and containing a significantly higher fraction of codons that have experienced selection for recurrent replacement of the encoded amino acid.

**Conclusion:**

Viruses may antagonize individual products of the interferon response more often than trying to neutralize the system altogether.

**Supplementary Information:**

The online version contains supplementary material available at 10.1186/s12862-021-01783-z.

## Introduction

The interferon response plays an important role in defending human cells against viruses [1]. Because viruses replicate within cells of the host, their nucleic acids and proteins are exposed, at least to some degree, to the cellular environment. To exploit this vulnerability of viruses, hosts have evolved numerous intracellular sensors that recognize viral nucleic acids and proteins [1, 2]. When cellular sensors detect virus-specific structures, a signaling cascade is activated which ultimately leads to the production and secretion of one or more interferon proteins [3, 4]. Interferons then produce transcriptional changes in the infected cell, inducing expression of hundreds of host genes (called “interferon-stimulated genes,” or ISGs) that collectively act to limit viral replication [5]. The resulting interferon-stimulated proteins act through a diversity of mechanisms [1, 5]. Additionally, interferons don’t just cause these transcriptional changes in the infected cell; they also signal to neighboring cells, even those that are uninfected, and induce the same transcriptional changes in those cells [6, 7]. In solid tissues, this produces a firewall of protected cells around the infected ones, impeding cell-to-cell spread of the virus.

Interferons use cell-surface receptors to signal and are organized into three classes based on the cell-surface receptor that they use. Type I interferons bind to the interferon α receptor (IFNAR), Type II interferons bind to interferon γ receptor (IFNGR), and Type III interferons bind to the interferon λ receptor (IFNLR) [8]. There are 21 unique genes encoding interferon proteins in the human genome. It remains unclear why there are more interferon proteins than receptors. Different interferon-receptor pairs may result in different signaling and transcriptional induction, but this is still an active area of research [3].

Viruses are formidable antagonists of the interferon response, and use diverse tactics to degrade, mislocalize, inhibit, or otherwise thwart proteins involved in interferon responses [9]. Viruses are known to target proteins that are both up- and downstream of the production of interferon molecules themselves [3]. As an example, STING is an important component of the signaling pathway leading to interferon production. Many flaviviruses encode proteins that target the host protein STING for degradation or inactivation [10–14]. Some of the same flaviviruses that inactivate STING also inactivate interferon-stimulated transcription factors (e.g., STAT1 and STAT2) as a second measure to fully ensure the interferon response is disabled [15–21].

Interferon pathways thus constitute a hotbed of antagonistic interactions between hosts and viruses. There is evolutionary pressure on viruses to evade or inhibit the interferon response, and then reciprocal pressure on the host to retain the advantage. This mode of evolution has been analogized as an “arms race,” in that it is ongoing with both parties evolving in response to the other. Host–pathogen arms races are responsible for the massive complexity of our immune system [22–26]. Indeed, many genes in mammalian interferon pathways have been identified as evolving rapidly, consistent with pathogens exerting selection for these proteins to alter key binding interfaces. Specific examples of proteins in the interferon pathway evolving under positive selection include cGAS, OASs, STING, and SAMHD1 [27–29]. In Table 1, we have listed some of the other examples of interferon-related genes that are under positive selection, as well as viral interactions that could be responsible. While there are many individual examples, a holistic understanding of whether different parts of the interferon response are evolving under different pressures has yet to be obtained. We provide such an analysis here. This analysis is important because identifying mammalian genes under positive selection, in particular if those genes are relevant to virus replication or defense, has proven itself to be a powerful shortcut in identifying host proteins that interact differently with viruses in one possible animal host versus another [26]. These are the host genes, therefore, that limit virus spillover between species [10, 23, 26, 30–42].

**Table 1:** Some examples of genes in Type I interferon pathways that bear the signature of successive rounds of positive natural selection

Category	Gene under positive selection	Known direct virus interactions	Literature showing positive selection
induction	MB21D1/cGAS	Many classes of viruses	Mozzi et al. 2015, Ma et al. 2016
Induction	IFI16	HCMV	van der Lee 2017, Dell’Oste et al. 2014
Induction	ISG15	Influenza	Zhao et al. 2013, Zhao et al. 2010
Induction	MAVS	Hepatitis C virus	van der Lee 2017, Anggakusuma et al. 2016
Induction	STING	Flaviviruses	Mozzi et al. 2015, Stabell et al. 2018, Ding et al. 2018
Induction	TRIM25	Influenza	Malfavon-Borja et al. 2013, Gack et al. 2009
ISG	ADAR	RNA viruses	Forni et al. 2014, Pfaller et al. 2018
ISG	MxB	Many classes of viruses	Mitchell et al. 2015, Haller et al. 2011
ISG	EIF2AK2/PKR	Influenza	Elde et al. 2009, Dauber et al. 2009
ISG	RNAse L	TMEV	van der Lee 2017, Sorgeloos et al. 2013
ISG	Tetherin	HIV	Lim et al. 2010, McNatt et al. 2009
ISG	TRIM15	Retroviruses	Malfavon-Borja et al. 2013, Uchil et al. 2008
ISG	TRIM22	Influenza	Sawyer et al. 2007, Di Pietro et al. 2013
ISG	TRIM31	Retroviruses	Malfavon-Borja et al. 2013, Uchil et al. 2008
ISG	TRIM38	Retroviruses	Malfavon-Borja et al. 2013, Uchil et al. 2008
ISG	TRIM5α	HIV	Johnson et al. 2009, Sawyer et al. 2005
ISG	RSAD2/Viperin	RNA viruses	Lim et al. 2012, Panayiotou et al. 2018
ISG	SAMHD1	HIV-2	Laguette et al. 2012, Coquel et al. 2018

## Results

### Curation of multiple sequence alignments

**Fig. 1:**
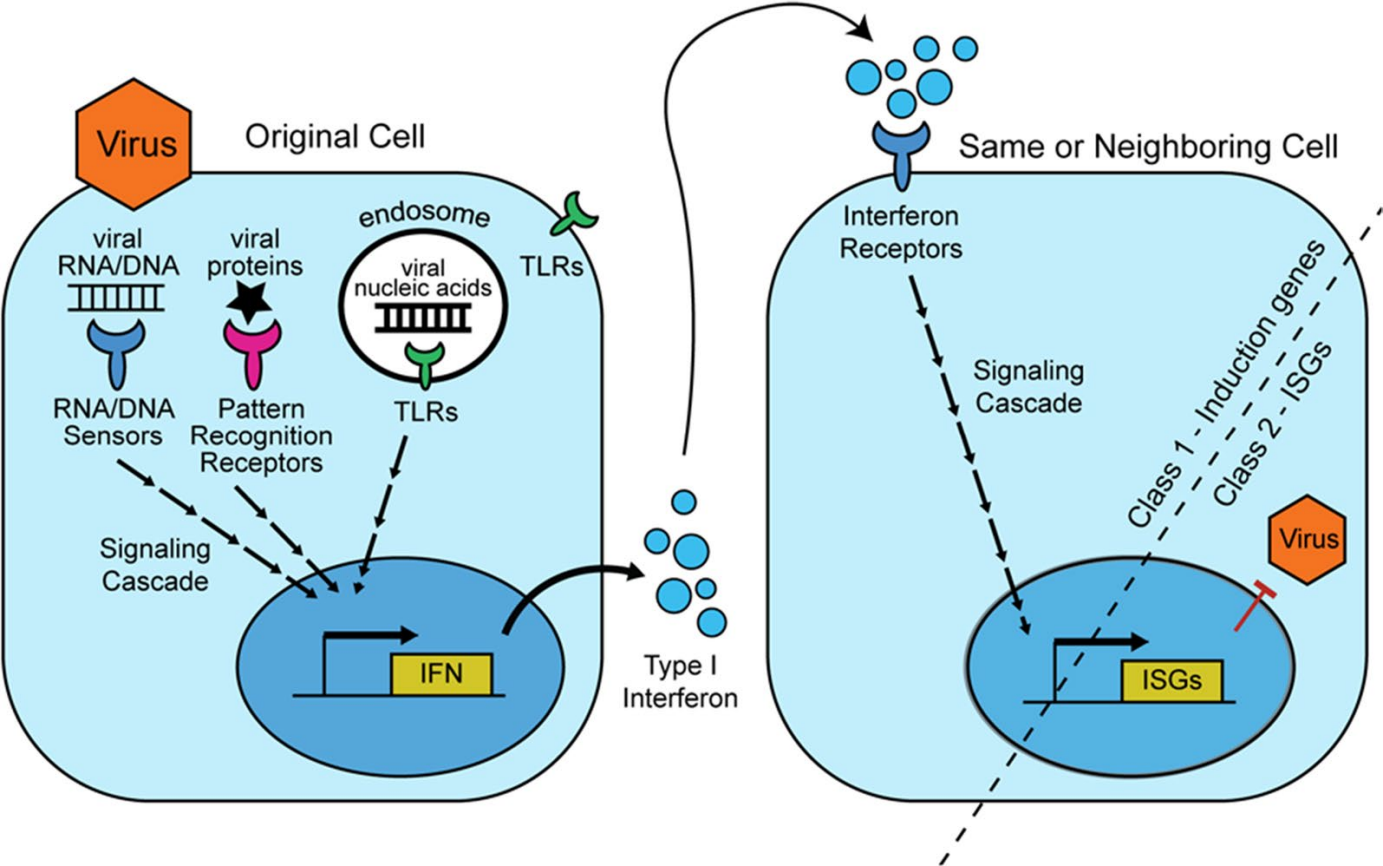
Definition of the gene classes analyzed in this study. A highly simplified illustration of the Type I interferon response is shown, to represent the two classes of genes analyzed. An infected, interferon-producing cell is shown on the left, and on the right is a cell then responding to the secreted interferon. In this study, “induction genes” are genes encoding any protein that acts in a way that ultimately leads to the expression of interferon-stimulated genes. Induction genes encode sensors of initial infection (pattern recognition receptors, toll-like receptors, and nucleic acid sensors), signaling cascade proteins, interferon molecules, interferon receptors, and transcription factors acting to induce interferon-stimulated genes. Also included are signaling molecules in the response to the interferon molecules that are produced and secreted (right cell). The second gene class, the interferon-stimulated genes, are a hugely diverse group of genes upregulated when cells are activated by interferon signaling. A relatively small number of these genes have been functionally characterized, but many encode proteins that interact directly with viruses or inhibit cellular processes that can be hijacked by viruses during infection

First, we curated lists of genes for our analysis. We separated genes involved in Type I interferon responses into two temporal categories with the dividing line being the expression of interferon-stimulated genes (Fig. 1). The interferon “induction” category contains genes acting upstream of the production of interferon α and β molecules—for instance, genes encoding proteins that identify pathogens, signal, and ultimately produce secreted interferon molecules, as well as the proteins that act downstream of interferon receptors and ultimately lead to the induction of interferon-stimulated genes. Interferon-stimulated genes are those that become expressed or over-expressed in the presence of interferon. We reviewed recent literature and curated lists of between 100 and 150 genes in each of these three categories: induction genes, interferon-stimulated genes, and random genes (using a random gene generator). In order for the evolution of a gene to be assessed, alignments of orthologous gene sequences are analyzed to quantify how substitutions have occurred over time. As such, simian primate orthologs of each of the induction, interferon-stimulated, and random genes were collected from GenBank and used to make a multiple sequence alignment for each gene. After visually inspecting and curating all alignments (see methods for alignment and quality control pipeline), we ended up with high quality multiple sequence alignments for 131 interferon-induction genes, 100 interferon-stimulated genes, and 100 random genes. The lists of genes analyzed can be found in Additional file 1.

**Fig. 2:**
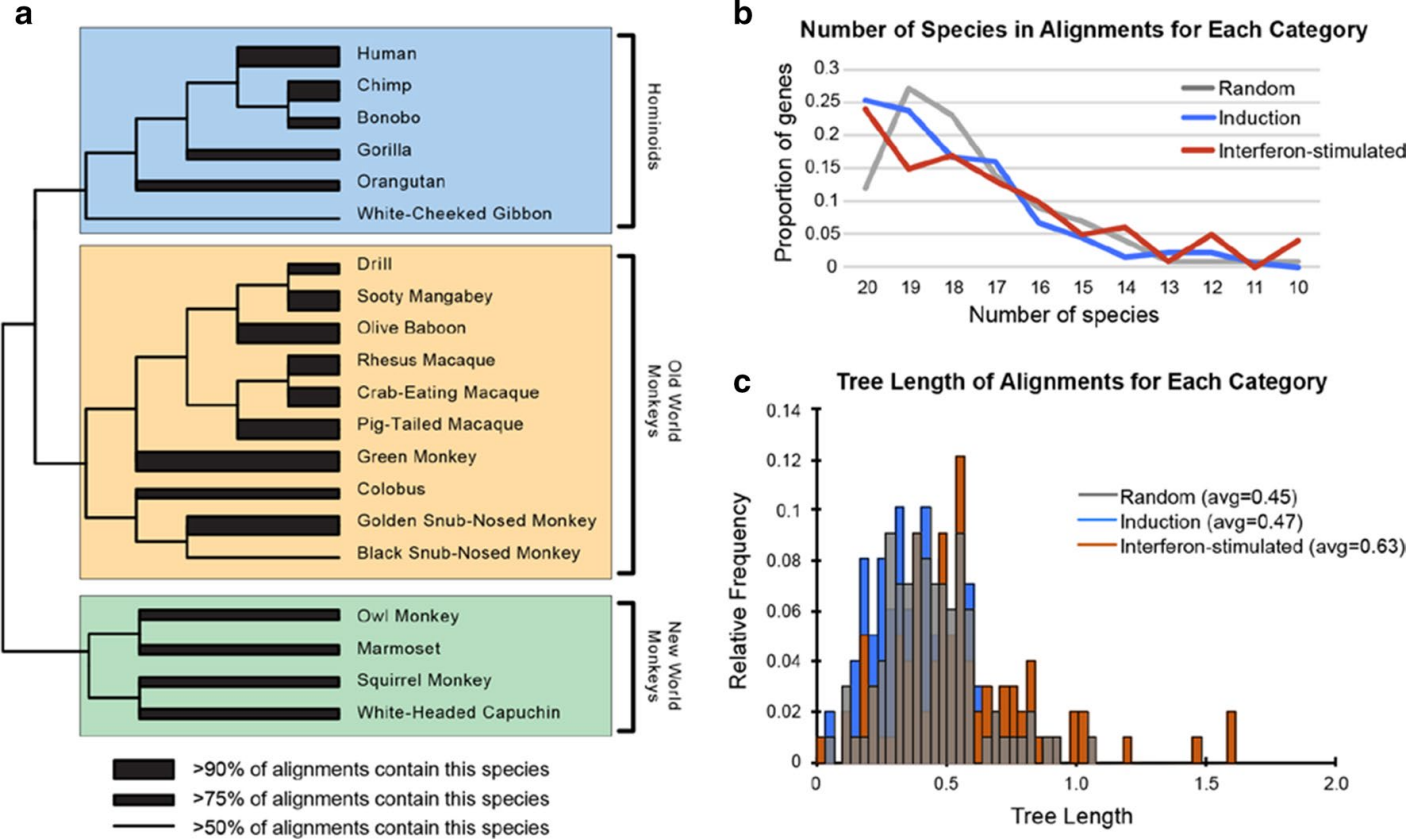
Quality and equity metrics for the three groups of multiple sequence alignments created. **a** A cladogram representing a species tree of the primates used in this analysis [44]. For each species, the branch width represents the percentage of multiple sequence alignments produced (out of 331) that contain that ortholog. All species were represented in over 50% of the alignments. Only white-cheeked gibbon and black snub-nosed monkey were represented in fewer than 75% of the alignments. **b** The number of species/sequences represented in the final 331 multiple sequence alignments, illustrated for each of the three categories of genes. **c** Tree length is the sum of the branch lengths along the tree or, in other words, the average number of nucleotide substitutions per site in an alignment. The relative frequencies of lengths are plotted as a separate histogram for each category, and the average tree length of each category is indicated in the legend

Because our goal is to compare evolutionary signatures between each of the three categories of genes, we wanted to confirm that the three datasets were similar in other qualities. First, we assessed the species composition of the datasets. A cladogram is shown of the species from which all sequences derive. Branch width demonstrates the percentage of the 331 multiple sequence alignments in which each species is represented (Fig. 2a). We then plotted the number of species represented in the alignments, for each of the three classes of genes. There are similar distributions of species in all three categories of datasets (Fig. 2b). We also compared the tree lengths of the multiple sequence alignments in each of the three categories. Tree length is a measure of sequence divergence and is the average number of nucleotide substitutions per site in a multiple sequence alignment [43]. The interferon-stimulated gene category appears to have alignments with tree lengths of greater value than either the induction or random gene sets (Fig. 2c). This was an initial hint that interferon-stimulated genes may be more genetically divergent than either induction genes or random genes. We next analyzed these datasets for signatures of positive selection.

### Interferon-stimulated genes experience more intense positive selection than interferon induction genes or randomly-selected genes

We analyzed each of the 331 multiple sequence alignments for signatures of recurrent positive natural selection [45–47]. Selection can operate on nonsynonymous substitutions (changing the encoded amino acid) and on synonymous mutations (silent, not changing the encoded amino acids). Most genes experience purifying selection, where non-synonymous mutations are strongly selected against over evolutionary time [23]. In contrast, gene regions or specific codons that have experienced repeated rounds of natural selection *in favor of* protein-altering mutations exhibit a characteristic inflation of the rate of non-synonymous (dN) DNA substitutions compared to synonymous (dS) substitutions (dN/dS > 1). Because nonsynonymous mutations occur more often than synonymous mutations by chance (due to the structure of the genetic code), computational models have been developed that use statistical frameworks to account for these unequal substitution rates [43, 48]. To evaluate patterns of dN/dS in these alignments, we used the statistical program Phylogenetic Analysis by Maximum Likelihood (PAML) [43]. PAML fits multiple sequence alignments to different models of codon evolution and calculates the likelihood of this model given the alignment data and known species tree.

**Fig. 3:**
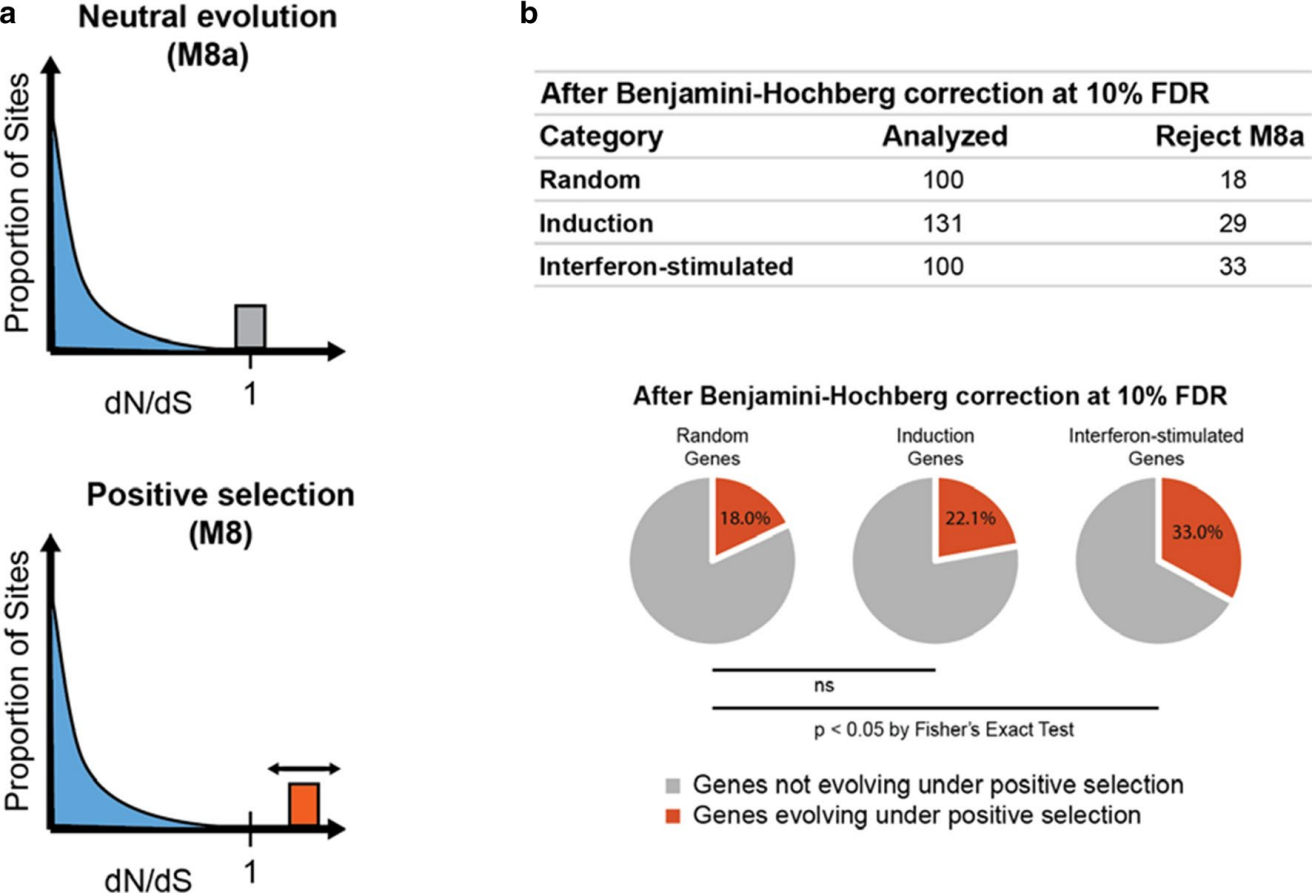
Interferon-stimulated genes are enriched for sequence signatures of recurrent positive natural selection. **a** Graphical illustrations of the M8 and M8a nested codon models in PAML (Yang 2007). M8a is a null model where all codons in the multiple sequence alignment must be placed into one of 11 bins of specific dN/dS values. Ten of these bins are distributed along a beta distribution of dN/dS values bounded between 0 and 1. The 11th bin is defined to have a dN/dS value = 1. M8 is identical, except that the 11th bin can have a dN/dS value greater than one [43]. The double-sided arrow indicates that the dN/dS value of this bin is optimized in the fitting of the data to the model. **b** 331 gene alignments were fit to both the M8 and the M8a models. A likelihood ratio test of the two nested models was conducted, and the final column indicates the number of genes in each category for which the null model M8a could be rejected in favor of the model of positive selection (p < 0.05). We did a Benjamini–Hochberg correction at 10% FDR to control for false positives. In the pie charts, the proportion of genes in each category that are under positive selection (red) is shown, from the table above. Using a two-tailed Fisher's exact test and Benjamini–Hochberg correction at 10% FDR, the difference in the number of genes rejecting the neutral model (M8a) between random genes and interferon-stimulated genes was significant

We first wanted to determine if any of our 331 genes are under positive selection and, if so, how this varies between the three categories of genes. We fit each alignment to two codon models, M8 and M8a, which are illustrated in Fig. 3a. M8a is a null model where all codons in the multiple sequence alignment must be placed into one of 11 bins of specific dN/dS values. Ten of these bins are distributed along a beta distribution of dN/dS values bounded between 0 and 1. The 11th bin is defined to have a dN/dS value = 1. M8 is identical, except that the 11th bin can have a dN/dS value greater than one [43]. Once a likelihood is determined for the data being represented by each of these models, a Likelihood Ratio Test is used to determine whether the null model (M8a) should be rejected in favor of the model allowing for positive selection of some codons (M8). After this analysis was performed on all 331 multiple sequence alignments, we ran the Benjamini–Hochberg procedure with an FDR of 10% to help correct for multiple testing and avoid false positives.

We found between 18 and 33 genes in each of the three categories to be under positive selection after Benjamini–Hochberg correction with an FDR of 10% (Fig. 3b). The number of genes under positive selection in the interferon-stimulated category was significantly larger than the number of genes under positive selection in the random category (two-tailed Fisher's exact test, p < 0.05; Fig. 3b). To ensure that this conclusion did not change when the number of genes in each category was equal, we chose 100 induction category genes randomly 100 times and compared the proportion of genes under positive selection in this category to the proportion of genes under positive selection in the interferon-stimulated and random categories for each instance. Ninety-two times out of 100, our results were the same and the number of genes under positive selection in the interferon-stimulated category was significantly larger than the number in the random category. In eight instances, the number of genes under positive selection in the interferon-stimulated category was significantly larger than the number in both the random and induction categories. We have included this analysis in Additional file 1. The genes identified as being under selection are listed in Table 2.

**Table 2:** Genes identified as evolving under positive selection

Induction genes evolving under positive selection	Interferon-stimulated genes evolving under positive selection
CASP10	ADAR*^†^
CIITA*	APOBEC3F^†^
CISH	APOBEC3G*^†^
DDX58*^†^	APOL2*
DDX60*^†^	APOL6*
EPOR	BST2*^†^
IFI16*	CCL8
IFNAR1*	CD47
IFNAR2	CEACAM1^†^
MAVS*^†^	CRP
MB21D1*^†^	DAPK1
MNDA	EIF2AK2*†
OAS1*^†^	GBP2*^†^
OAS2*^†^	IFI27^†^
PTPRC^†^	IFI44
PYHIN1	IFI44L
RNASEL*^†^	IFI6
SPP1	IFIT1*
STAT2*^†^	IFIT2*
TLR1*^†^	MLKL
TLR2*^†^	MX1*^†^
TLR4*^†^	MX2*^†^
TLR5*^†^	PHF11
TLR6*^†^	RSAD2*^†^
TLR8*^†^	RTP4*^†^
TMEM173*^†^	SAMD9
TRIM21*^†^	SAMHD1*^†^
TRIM25*^†^	SLFN12*
TYK2	TAGAP
	TMEM140
	TNK2
	TRIM22*^†^
	TRIM5*^†^
	ZC3HAV1*^†^

Genes that have been previously identified as rapidly evolving in primates (*) and which genes have known interactions with pathogenic elements (^†^) are from the following studies: [27, 42, 49–64]

* Previously identified as being under positive selection in primates

^† ^Published interaction with pathogen

**Fig. 4:**
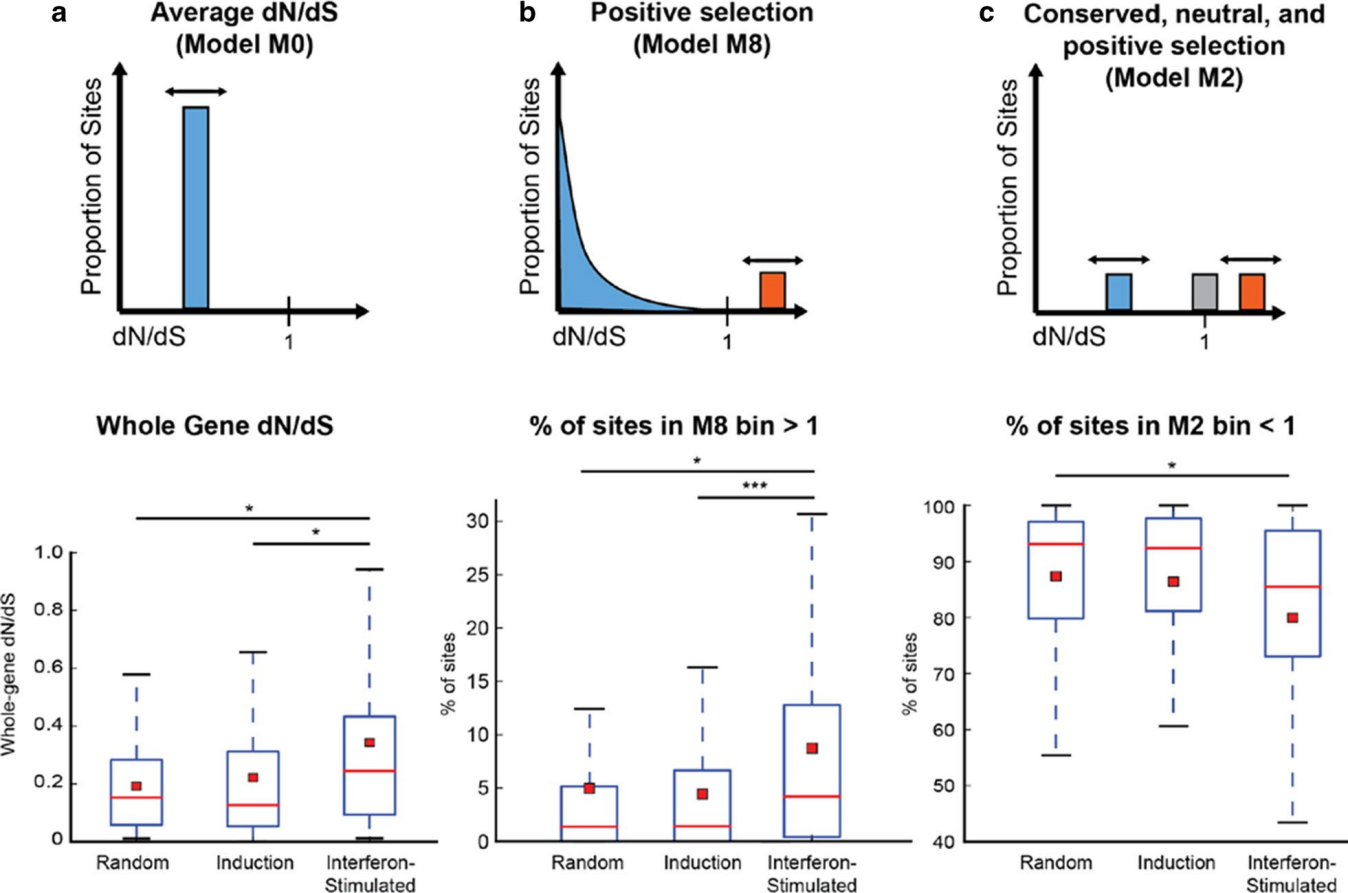
Interferon-stimulated genes have a higher whole-gene dN/dS value and more codons under positive selection than other genes.** a** Top: M0 is a codon model in PAML where all codons in an alignment are assigned to a single estimated dN/dS value. Below: box plot of the whole gene average dN/dS values determined by M0 in each category. *p-value < 0.05. **a** Top: The M8 model of codon evolution, as explained in the legend to Fig. 3a. Below: box plot of percentage of codon sites per gene in the dN/dS > 1 bin in the M8 model. *p-value < 0.05; ***p-value < 0.001. **C** Top: M2, a simple model that allows for positive selection, places all codons into one of three bins: a bin at dN/dS < 1 (conserved), a bin at dN/dS = 1 (neutral), and a bin at dN/dS > 1 (positive selection). The double-sided arrow indicates that the dN/dS value of this bin is optimized in the fitting of the data to the model. Below: box plot of the proportion of codon sites per gene in the dN/dS < 1 bin in model M2. *p-value < 0.05

We next evaluated whether the intensity of selection might be different between these gene categories by utilizing other codon models in PAML. We compared the whole-gene (i.e. global) dN/dS values estimated for each multiple sequence alignment, calculated using the PAML model M0 (Fig. 4a, top). M0 is a model that allows only a single bin, with an optimized value of dN/dS, into which all codons must be placed. The average whole-gene dN/dS values for interferon-stimulated genes were significantly different than both those for our random set and the interferon induction set (Kolmogorov–Smirnov test; Fig. 4a, bottom). This suggests either that interferon-stimulated genes are evolving more rapidly than the other genes, or are experiencing more neutral evolution.

To differentiate between these two possibilities, we looked more closely at the codons assigned to the dN/dS > 1 bin in M8. We tested whether a higher percentage of codons were placed in the M8 bin > 1 for interferon-stimulated genes than for genes in the random or induction categories. Indeed, the average percentage of codons that fell in the estimated M8 dN/dS > 1 bin was significantly greater for interferon-stimulated genes (Fig. 4b). This suggests that interferon-stimulated genes are, on average, experiencing more positive natural selection than genes in the other two categories.

If more codons are under positive selection, we might expect fewer codons would be evolving under negative selection (i.e., conserved in sequence). M2, a simple model that allows for positive selection, places all codons into one of three bins: a bin at dN/dS less than one (conserved), a bin at dN/dS = 1 (neutral), and a bin at dN/dS > 1 (positive selection) (Fig. 4c, top). We tested whether PAML placed fewer codons in the M2 bin of dN/dS < 1 for interferon-stimulated genes then for genes in the induction or random category. Indeed, for interferon-stimulated genes the average percentage of codons placed in the M2 bin less than 1 is significantly less than for random genes (Fig. 4c, bottom). This is consistent with interferon-stimulated genes having fewer codons under negative selection (i.e., constraint).

## Discussion

We find that interferon-stimulated genes are evolving more rapidly than canonical interferon induction genes and more rapidly than our sample of random human genes. Rapidly evolving host genes are key in enforcing species barriers to viral spillover [26]. While the entirety of the human immune system is important, only the parts that are functionally divergent from the immune systems of other animals are important in the defense against zoonotic viruses. In other words, any immune obstacle that a virus has already overcome in an animal will not be a barrier to infecting humans unless that obstacle has taken on a different flavor of interaction in the human genome. Arms races are the selective engine that drive rapid sequence evolution at the interaction interfaces between host and virus proteins, as they each jockey to establish or destroy these interactions [65]. This conflict matters because it means that these interactions play out differently in different species, and thus these evolutionary dynamics enforce species barriers to the transmission of pathogens.

We had hypothesized that viruses are more likely to evolve mechanisms to halt the production of antiviral cellular states by antagonizing the initial expression of interferons rather than the individual proteins which produce antiviral states. Intuitively, it makes sense to “turn off the tap” instead of trying to mop up the after-effects of an induced interferon response. Therefore, we expected that the induction pathway would be under greater pressure to evolve rapidly and that we would see a higher signal of positive selection in the induction pathway. Instead, we found that interferon-stimulated genes are evolving more rapidly than both a randomly drawn set of human genes and proteins involved in interferon induction. We have not made conclusions about the strength of positive selection on specific sites or genes in any of these categories—rather, we have analyzed these genes as groups and found significant differences between interferon-stimulated genes and the other two categories. It is possible that induction genes as a whole are under more evolutionary constraint in order to preserve specific functions in their respective signaling pathways. Previous work suggests that interferon genes themselves are evolving under different evolutionary constraints [8]. The interferon molecules and receptors, though much expanded in extant species, is an ancient class of proteins that has had to evolve under the constraints of binding partner compatibility [66]. We show that the interferon receptors are evolving under positive selection, but interferons and interferon-signaling genes may, as a class, be more constrained. In contrast, interferon-stimulated genes may have more flexibility to obtain and tolerate mutations. Because interferon-stimulated genes are sometimes more specific in function or can rely on the redundancies of the induced response, nonsynonymous mutations may be tolerated to a greater extent. For example, mammalian cells have several ways to shut down host and virus translation during viral infection: the IFIT family of proteins, ISG15, and ZAP are all examples of proteins that are induced by interferon and prevent viruses and hosts from translating RNA [67, 68]. Redundancy in this specific antiviral defense might mean that mutations can be more easily tolerated in each individual protein. Interferon-stimulated genes remain relatively understudied in terms of their mechanistic antiviral action [69]. However, they may be at the forefront of the host-virus “arms race” that has implications for pathogenicity of viruses, the ability of viruses to spillover to new hosts, and the evolution of our immune systems. Finally, we note that although we approached the curation of these genes with a particular emphasis on genes important in antiviral pathways, many of the genes that have been analyzed here are involved in the immune system’s defense against other types of pathogens as well. Therefore, the positive natural selection we have identified partly reflects antagonism by other types of pathogen.

## Methods

### Definition of gene categories

The list of 131 interferon induction genes was curated from reviews of interferon signaling pathways [70–74]. We didn’t include genes listed in these reviews that were primarily belonging to DNA damage pathway, since these pathways have been shown to experience positive selection as well [75–77]. We do not assume that this “induction” category is a complete list of genes upstream of interferon-stimulated genes expression, but rather have treated it as a representative list of genes known to be implicated in several induction pathways. Any gene mentioned in the reviews that could not be unambiguously identified (i.e. gene name was listed as an alias for multiple genes) was removed from this list. The list of 100 interferon-stimulated genes was curated from published literature [5, 78–80]. These genes were verified by the *Interferome* database [81], with the criteria that each interferon-stimulated gene was upregulated at least twofold by Type I interferons. A list of random human genes was formed using a random gene set generator [82]. We did not place the same gene in more than one category. If a gene is implicated in canonical induction pathway, but also upregulated by interferons, it was placed in the induction category.

### Creation of multiple sequence alignments for each gene

The longest human isoform of each gene, along with any simian primate orthologs available, were collected from the NCBI Gene database. We collected and retained as many primate sequences as possible, including sequences that were labeled as unassigned gene loci, as long as that sequence returned the correct human ortholog in a reciprocal BLAST search back to the human genome. In some cases, primate orthologs contained “n”s suggesting that these bases did not meet certain quality thresholds. These sequences were retained, but note that PAML treats “n’s” as gaps and will therefore not analyze codons in multiple sequence alignments that contain them. Further, any sequence that was marked holistically as “Low Quality” on NCBI were not included. The cDNA sequences were then translated to amino acids and aligned with the MUSCLE algorithm using the Unipro UGENE software [83] or MEGA [84]. Pal2Nal [85] was then used to reference this amino acid alignment while producing a final alignment of cDNA by codon. The result was over 300 multiple sequence alignments containing human and primate orthologs of interferon-related or random genes.

Each multiple sequence alignment was then manually inspected and edited. Our pruning and quality control pipeline consisted of these steps. (1) We removed from the alignments any ortholog containing a gap (missing sequence) that spanned > 10% of the length of the cognate human gene. This was done because PAML won’t analyze codon sites containing a gap. We did not remove an ortholog if it had multiple gaps relative to the human sequence, as long as each gapped region was < 10% of the length of the human gene. (2) We removed from the alignments any ortholog which aligned poorly to other sequences in the alignments for a contiguous stretch spanning > 10% of the length of the human gene. We did this because simian primate sequences tend to align with very high identity since divergence is low in this clade [86], and such regions usually indicate regions of mis-annotation or gene prediction. (3) We trimmed from alignments sequence at either terminal end (starting at start codon and ending at stop codon) if less than ten orthologs in the alignment had the same start or termination site as the human sequence. In this case, we stopped trimming alignments at the first conserved site (or site where amino acid variation tracked with phylogeny). (4) We manually inspected all remaining gaps in the multiple sequence alignments. We deleted codon columns where more than one amino acid misaligned at the edge of a gap. (5) We deleted all regions in the alignments where an ortholog contained more than four amino acids in a row that did not align to any other orthologs in the alignment. (6) After all of these curation steps, multiple sequence alignments containing less than 10 orthologs (including human) were not analyzed further. This is because we have previously shown that the accuracy of evolutionary tests improves as the number of primate species and overall tree length of an alignment increases [86].

### Evolutionary analysis

Positive selection was detected using the Phylogenetic Analysis by Maximum Likelihood (PAML) program. The *codeml* program packaged in PAML accommodates for the differences in rates of transition/transversion substitutions, unequal codon frequencies, and the probabilities of mutation across the codon [43]. PAML requires the codon alignment be accompanied by a phylogenetic tree to accurately identify rates of substitutions. A master phylogenetic tree with the twenty possible primate species was made using Perelman et al. 2011 as a reference and modified as necessary for each gene [44]. In all cases except in the fitting of data to the M8a model we kept the default parameters of the codeml program for each model and allowed omega to be estimated. In fitting data to the M8a model we fixed omega at 1. The tree length of each multiple sequence alignment was determined from the output file of model M0.

PAML fits the multiple sequence alignments to different models of codon substitution [43]. For the analysis outlined in this paper, we used the M0, M2, M8a, and M8 models. We used likelihood ratio tests to determine which model, M8 or M8a, best fit the data for the evolution of each gene. PAML provides a log likelihood (lnL) value for each alignment in both the null and positive selection models. The difference of these values is then doubled, referred to here as “2ΔlnL”, and used to perform Chi-Square tests with a single degree of freedom. We defined a p-value of p < 0.05 allowing us to reject the null hypothesis that there is no difference in how well models M8 and M8a fit the data. These genes were determined to be evolving under positive selection.

In instances where M8a was rejected in favor of M8, specific codons are identified which have elevated rates of nonsynonymous fixed mutations. This is determined by the Bayes empirical Bayes (BEB) method which accounts for sampling errors in the parameters of the model [43]. The codons identified by BEB, and the posterior probability by which they are predicted to fall in the bin > 1, was recorded (Additional file 1).

## Supplementary Information

**Additional file 1.** Summary of genes analysed in this study with evolutionary model information.

## Data Availability

The datasets produced and/or analyzed during the current study available from the corresponding author on reasonable request.
